# Digital Health and Climate Change

**DOI:** 10.1055/s-0046-1816598

**Published:** 2026-11-03

**Authors:** Linda W. Peute, Romaric Marcilly

**Affiliations:** 1 Amsterdam UMC, Univ. of Amsterdam - eHealth Living & Learning Lab Amsterdam, Department of Medical InformaticsAmsterdamNetherlands; 2 Univ. Lille, CHU Lille, ULR 2694 - METRICS : Évaluation des technologies de santé et des pratiques médicalesF-59000 LilleFrance; 3 InsermCIC-IT 1403, F-59000 LilleFrance

**Keywords:** Digital Health, Climate Change, Telemedicine, Carbon Footprint

## Abstract

**Objective:**

To give an overview of recent research and propose a selection of best papers published in 2024 on digital health and climate change.

**Methods:**

A comprehensive search of PubMed and Web of Science was conducted to identify peer-reviewed articles published between December 2023 and December 2024 on the theme “Digital Health and Climate Change”. Titles and abstracts were screened independently by the two editors to identify candidate papers for the best paper selection process. This process included: (1) a detailed review of the shortlisted papers by the two editors, six experts from internationally recognized research groups, and the Yearbook editorial committee; and (2) the final selection of the best paper during an in-person consensus meeting of all Yearbook editors.

**Results:**

The selected papers showcase advances in using digital health to support climate goals, with most focusing on telemedicine’s role in reducing greenhouse gas emissions. Selected studies largely fall into two categories: (1) the design of digital technologies aimed at climate resilience, and (2) the environmental effects of digitally supported care models, especially telemedicine.

**Conclusion:**

Telemedicine emerges as a key enabler of sustainable healthcare in 2024, while broader and more holistic evaluations remain essential for future progress.

## 1. Introduction

The integration of sustainability into digital health research has gained momentum in 2024, reflecting a broader global imperative to reduce the environmental impact of healthcare systems. The selected articles for this special topic converge on a common theme: the role of telemedicine in reducing greenhouse gas (GHG) emissions, primarily by minimizing patient travel. Across the body of work reviewed, teleconsultation consistently emerges as a promising strategy to align health service delivery with climate goals, while maintaining or even enhancing access to care.

Most studies adopt a quantitative lens, estimating avoided emissions when in-person visits are replaced by remote consultations. These analyses vary in scale, from national-level assessments to specialty-specific scenarios. Together, they present a coherent message: the environmental benefits of telehealth are substantial, particularly in contexts where long travel distances are common. Beyond carbon savings, some studies begin to explore additional dimensions, such as economic implications and care accessibility, signaling a shift toward multidimensional sustainability assessments.

Despite this progress, several gaps appear. Only a minority of studies account for the emissions generated by the digital infrastructure supporting telemedicine, and very few of the selected articles adopt life cycle or holistic approaches that capture both direct and indirect impacts. Evidence outside high-income settings is still scarce, even though these regions stand to gain significantly from resource-efficient innovations. Furthermore, clinical outcomes are rarely considered alongside environmental metrics, raising questions about how sustainability goals can be achieved without compromising quality of care. Collectively, the selected papers in 2024 on digital health and climate change illuminate the current progress of sustainability-focused research in (bio)medical informatics.

## 2. Methods

We conducted an extensive literature search following a process commonly used by IMIA Yearbook section editors. The search was carried out on MEDLINE and Web of Science. We have deliberately built a fairly high-level query to cover a wide range of digital technologies and to avoid incurring excessive constraints. The query has two components: “digital health”, ‘telehealth’, “health technology” on the one hand and “climate change” on the other hand.

((digital health OR telehealth OR health technology) AND (climate change)

All terms except “digital health” are retrieved from the MeSH thesaurus. The query was performed in January 2025. Only original research published in English between December 1, 2023 and December 31, 2024 in peer-reviewed scientific journals were considered; we excluded the various types of literature review, editorials, letters to the editor, or conference proceedings.

The paper selection was performed and reported following the recommended PRISMA guidelines. Once the queries had been performed, the results from both databases were compiled in a unique spreadsheet. Duplicate articles were identified and removed. The two section editors then independently screened the titles and abstracts of the remaining articles to identify and retain those corresponding to the inclusion criteria. All articles retained after title and abstract screening were fully read by both section editors independently to decide on their final eligibility. At each stage, disagreements over eligibility were discussed between the two editors until a consensus emerged. The process was over-inclusive: in case of doubt, the article was included in the next screening stage. Papers published in previous editions of the IMIA Yearbook, as well as unavailable articles, along with papers that were co-authored by one of the section editors were also excluded.

Papers meeting the eligibility criteria were independently re-read by the section editors and classified into two categories (shortlisted or not) based on their innovativeness, scientific and practical impact, and methodological quality. Each researcher wrote a note justifying their decision. These decisions were finally discussed together until both section editors agreed on the shortlist. In line with the IMIA Yearbook selection process, papers included in the shortlist were evaluated and rated by six independent reviewers, experts in medical or public health informatics, the two section editors and the chief editors. The selection of the “best papers” was then discussed in an in-person consensus meeting with all editors of the Yearbook, based on the score obtained during this last evaluation, as well as on the opinion of the section editors (based on the article’s interest for the community, its scientific rigor, and its innovation).

## 3. Results

### 3.1 Review Process and Shortlist

The MEDLINE query identified 377 articles, and Web of Science 35, giving a total of 432 articles. Of these, 20 were removed because they duplicated other articles. No additional records were identified through other sources. The first screening stage involved 392 articles; Out of the 392 records undergoing an initial screening, 340 were excluded because their titles or abstracts did not adequately match to the terms used in the query. As a result, 52 articles were read in their entirety and checked against the eligibility criteria. 23 met them. Of these 23, 12 were shortlisted, and two of them were awarded best papers. The 12 shortlisted articles address two main themes: (1) the design of digital technologies with respect to climate change (2 articles), and (2) the environmental impact of digitally supported care organizations, primarily teleconsultation (10 articles). This focus reflects current research trends, which are heavily concentrated on telemedicine and its potential for sustainability.

#### Digital Technology and Climate Change

These two articles differ in focus, one proposes a conceptual framework for designing digital technologies, while the other evaluates a specific technology. Iyengar *et al.* [ 1 ] introduce the concept of “Resilience Informatics” (RI), emphasizing the use of digital technologies (AI, sensors, messaging, mobile health) to strengthen the resilience of individuals and communities facing health, climate, or social crises. The authors present a six-step framework for developing effective, flexible RI solutions tailored to local contexts. This framework is illustrated through a case study. The article highlights the critical need for culturally relevant, accessible, and equitable design, especially in low-resource countries.

The study by Eggeling *et al.* [ 2 ] validates ClimApp, an application designed to predict individual heat stress in cold environments by combining local weather data with personal characteristics such as activity level, clothing, and body morphology. In a controlled trial, 55 participants were exposed to temperatures below 10°C for one hour and reported their thermal sensations, which were then compared to the app’s predictions. Results demonstrated that ClimApp outperformed the Universal Temperature Climate Index in accurately forecasting perceived thermal sensation. Beyond validating the app, the study underscores the importance of personalization in enhancing the effectiveness of heat stress prevention tools, especially amid increasing extreme weather events.

#### Impact of Telemedicine

Of the 12 shortlisted articles, 10 deal with the environmental impact of telemedicine, mainly in terms of GHG, especially carbon dioxide (CO2) emissions. Of these, three assess this impact at institutional or even state level, and seven focus on a particular pathology or medical specialty.

Among the three broadly focused articles, the retrospective observational study by Cummins *et al.* [ 3 ] stands out for its extensive scope. It analyzed the travel distances that 79,904 patients in the USA would have covered for in-person consultations if teleconsultations had not been available, alongside the associated GHG emissions. The authors also estimated emissions generated by telemedicine itself, including energy use for video conferencing and data transfer. Their findings indicate that for the USA as a whole, taking into account the emissions induced by the use of telemedicine tools during 2021, 1,443,800 tons of CO2 could nonetheless be saved thanks to telemedicine.

On a more local scale, Madison *et al.* [ 4 ] investigated the extent to which the increase in teleconsultations during the COVID-19 pandemic period affected the net GHG emissions of a rural hospital in New -Hampshire. As the hospital is located in a very rural area, patients often have to travel long distances to get there. The 251,832 teleconsultations carried out during three periods (pre-epidemic, early pandemic, late pandemic) were included. The distance travelled by patients if they had had to come by car was converted into kg of CO2. To calculate the net CO2 savings, the authors also estimated the carbon footprint of the teleconsultation according to the medium used (videoconference platform or telephone) and deducted it from the amount of CO2 saved. Because the distances travelled by patients during the three periods varied, so did the CO2 savings. The authors highlight that, especially in rural areas, net energy savings from telemedicine should be weighed alongside accessibility and cost considerations of care. In addition to large databases, these two papers [3,4] adopt an approach that takes into account both the GHG savings made by teleconsultation and the GHG costs it incurs.

The third large scale study by Moore *et al.* [ 5 ] addresses a less studied segment of telemedicine: the e-consultations between general practitioners and specialists, which enable asynchronous exchanges between primary care and specialists, thereby reducing the need for face-to-face visits in specialist care. This study examines the impact of an academic hospital’s e-consultation program on patient travel distances and the GHG reductions associated with distance reductions. To this end, the authors conducted a retrospective observational study analyzing electronic health record data of 11,590 complete e-consultations performed between July 2018 and December 2022. The results show that by eliminating unnecessary physical specialist consultations through asynchronous specialist advice, 205 tons of CO₂ were saved.

The seven other articles selected for the shortlist also look at the impact of telemedicine on GHG emissions, not from a state or facility level, but rather from a medical specialty point of view.

In the field of urology, Wurtz *et al.* [ 6 ] focus on pre-vasectomy consultations. Retrospectively analyzing the records of 126 patients, the authors show that each teleconsultation carried out in place of a physical consultation saves an average of 9 kg of CO₂. Extrapolated to the 500,000 vasectomies performed each year in the USA, this organizational change could avoid 50,000 tons of CO2.

In cardiology, the retrospective study by Gunn *et al.* [ 7 ] quantifies the CO2 and other GHGs not emitted during almost 15,000 teleconsultations carried out over 45 months with 9,942 patients. Their analysis shows that each remote consultation saves 10.06 kg of CO2. To make the data more meaningful, the authors compare the savings achieved over 45 months with the number of trees that would have had to be planted to offset this effect (9,000). Still in the cardiology field, Özen *et al.* [ 8 ] attempt to model the potential impact of telemedicine in three cardiovascular clinics in Istanbul. Analyzing 45,602 visits between 2015 and 2023, they show that replacing face-to-face follow-up consultations with virtual visits would save 656,000 km of travel, reducing CO2 emissions by 30%. At the same time, it would avoid nearly $504,000 in lost wages due to travel time. The study concludes that telemedicine is a lever for more sustainable health, while improving accessibility and social productivity. It should be noted that this study is the only one on the shortlist was not conducted in a high-income country, and that the lack of resources is the primary motivation for implementing telemedicine, while reducing CO2 emissions is more of a side-effect.

Obstetrics was also the subject of a targeted study: Li Valverde *et al.* [ 9 ] examined the savings made over 7 months in terms of kilometers traveled, fuel costs and gas emissions when consultations for high-risk pregnancies were carried out remotely. The results show that for the 2,454 teleconsultation visits carried out, 94.6 tons of gas were not emitted. The impact is particularly significant for patients who are farthest from their health-care center.

Although still at the level of a medical specialty, the last three articles stand out from the others either by their method (Heffernan *et al.* [ 10 ]), the level of analysis (Grüßer *et al.* [ 11 ]) or its breadth (Opperlaar *et al.* [ 12 ]). In the pediatric otolaryngology field, Heffernan *et al.* [ 10 ] seek to determine the carbon savings potential of incorporating virtual care into surgical care pathways for pediatric patients with obstructive sleep apnea or otitis. Where other studies have been retrospective, this study stands out for its use of a modelling cohort study design. The researchers developed care scenarios in which consultations were listed; they also defined the location of possible patients in the center of the two most populous municipalities in the 10 most populous districts of the British Columbia (BC, Canada) state. They simulate the effect of three virtual (rather than physical) visits for children with sleep apnea or ear infections. The potential savings are up to 2156,8 kg CO2 per child for a whole care scenario. This study underlines the important potential of teleconsultation for pathologies requiring repeated follow-up.

Of the shortlist of studies presented so far, only two had opted for a net CO2 saving calculation, the others focusing solely on emission reductions without taking into account the GHGs produced by the use of telemedicine. A third, focusing on anesthesia teleconsultations, also adopted this approach using a holistic approach. Grüßer *et al.* [ 11 ] assess the environmental impact of preoperative teleconsultations in anesthesia using a life cycle assessment. They compare two scenarios (face-to-face consultation vs. teleconsultation) based on data from 821 patients and anesthetists at Aachen University Hospital. The results show that for patients coming only for the anesthesia appointment, if this appointment is carried out remotely, 3 tons of CO2 emission can be saved from the first month. This figure could rise to 1,300 tons over 10 years if teleconsultations became widespread. The impact of manufacturing digital equipment is largely offset by the avoided journeys.

Finally, in line with the other studies presented, Oppelaar *et al.* [ 12 ] looked at the environmental impact of teleconsultation and telemonitoring for the specific case of cystic fibrosis patients. However, they dive deeper by also considering the clinical impact of teleconsultation. They compared data from 81 patients one year before COVID-19 with 2 years afterwards: in the year before COVID-19, consultations and spirometry tests were mostly carried out face-to-face, whereas in the 2 years afterwards, consultations were also carried out remotely, and spirometry tests were even more numerous remotely than face-to-face. The total reduction in GHGs over the two years of COVID-19 was 5,450.3 kg CO2 emission, which would represent 111,390.5 kg CO2 emission if extrapolated to all patients with cystic fibrosis in the Netherlands in 2022 (equivalent to 5,068 mature trees). Most importantly, this reduction in GHGs did not lead to any deterioration in the patients’ state of health, with respiratory function declining slightly, in line with the natural course of the disease. The study underlines that remote care can reduce the carbon footprint of the healthcare system without compromising clinical outcomes.

## 4. Conclusion

On the basis of ratings by experts and editors, and after discussion between section editors and chief editors, two articles published in 2024 were named best articles for this section (Table 1).

**Table 1:** Best paper selection of articles for the IMIA Yearbook of Medical Informatics 2025 in the special topic section ‘Digital Health and Climate Change’. The articles are listed in alphabetical order of the first author’s surname

**Selected best papers 2024 for the Special Topic “Digital Health and Climate Change”**
Cummins MR, Shishupal S, Wong B, Wan N, Han J, Johnny JD, Mhatre-Owens A, Gouripeddi R, Ivanova J, Ong T, Soni H, Barrera J, Wilczewski H, Welch BM, Bunnell BE. Travel Distance Between Participants in US Telemedicine Sessions With Estimates of Emissions Savings: Observational Study. J Med Internet Res. 2024 May 15;26:e53437. https://doi.org/10.2196/53437 . PMID: 38536065; PMCID: PMC11137427. Oppelaar MC, Bannier MAGE, Reijers MHE, van der Vaart H, van der Meer R, Altenburg J, Conemans L, Rottier BL, Nuijsink M, van den Wijngaart LS, Merkus PJFM, Roukema J. Remote monitoring and teleconsultations can reduce greenhouse gas emissions while maintaining quality of care in cystic fibrosis. Front Digit Health. 2024 Oct 24;6:1469860. https://doi.org/10.3389/fdgth.2024.1469860 . PMID: 39512858; PMCID: PMC11540799.

Concluding, the following articles have been selected as best papers.

The article by Cummins *et al.* [ 3 ] stands out from the others for its national scope, analyzing CO2 savings on a US-wide scale, unlike many more local or medical specialty-specific studies. Furthermore, it is one of the few to have adopted a global approach, not only studying CO2 savings but also investigating the CO2 cost induced by the use of teleconsultation. Its methodology is rigorous and clearly explained. Despite its complexity, the article proves invaluable for researchers in medical informatics and public health.

The article by Oppelaar *et al.* [ 12 ] stands out for its balanced approach, assessing both the environmental impact of teleconsultation and its impact on patients’ health status, giving it a more global and original perspective. The writing is clear and the retrospective methodology is rigorously detailed. Despite its small sample size, this article is an excellent illustration of an evaluation aimed at two very complementary objectives: reducing the carbon footprint of healthcare and maintaining a high level of healthcare quality.

Both papers are good illustrations of the current research trends in digital health and climate change. They offer a glimpse of the quality and breadth of work being conducted at the intersection of health, technology, and environmental stewardship. Future work will need to broaden its scope by integrating comprehensive environmental accounting, equity considerations, and clinical impact. This is needed to guide informed policy and implementation strategies at the intersection of health and sustainability.
